# Pemigatinib in the Real-World Management of Cholangiocarcinoma Through a Canadian Patient Support Program

**DOI:** 10.3390/curroncol32070405

**Published:** 2025-07-16

**Authors:** Philip Q. Ding, Vincent C. Tam, Ravi Ramjeesingh, Jamil Asselah, Brandon S. Sheffield, Taylor Mitchell, Anne-Julie Gaudreau, Jennifer J. Knox, Winson Y. Cheung

**Affiliations:** 1 Oncology Outcomes Research Program, University of Calgary, Calgary, AB T2N 4N1, Canada; philip.ding@ahs.ca; 2 Faculty of Medicine & Dentistry, University of Alberta, Edmonton, AB T6G 2R3, Canada; 3 Division of Medical Oncology, Department of Oncology, University of Calgary, Calgary, AB T2N 5G2, Canada; vincent.tam@albertahealthservices.ca; 4 Division of Medical Oncology, Department of Medicine, Nova Scotia Health, Dalhousie University, Halifax, NS B3H 2Y9, Canada; ravi.ramjeesingh@nshealth.ca; 5 Division of Medical Oncology, Royal Victoria Hospital, McGill University Health Centre, Montreal, QC H4A 3J1, Canada; jamil.asselah@mcgill.ca; 6 Division of Advanced Diagnostics, William Osler Health System, Brampton, ON L6R 3J7, Canada; 7 Incyte Biosciences Canada, Pointe-Claire, QC H9R 0A5, Canada; tamitchell@incyte.com (T.M.); anne-julie.gaudreau@astellas.com (A.-J.G.); 8 Department of Medical Oncology, UHN Princess Margaret Cancer Centre, University of Toronto, Toronto, ON M5G 2M9, Canada; jennifer.knox@uhn.ca

**Keywords:** cholangiocarcinoma, real world, pemigatinib, systemic therapy, response, survival

## Abstract

Background: In September 2021, pemigatinib received Health Canada approval for previously treated locally advanced/metastatic cholangiocarcinoma (CCA) with *FGFR2* rearrangements/fusions. This retrospective study aimed to characterize the real-world management and outcomes of patients with CCA receiving pemigatinib through a Canadian patient support program (PSP). Methods: We evaluated a multi-centre case series of Canadian patients who were prescribed pemigatinib between September 2021 and January 2023 for eligible CCA diagnoses and enrolled in the PSP. The retrospective study data included demographic and disease-, treatment-, and outcome-related information, and these were collected using a survey of prescribing physicians. Results: Of the 26 patients who initiated pemigatinib in the PSP, we received survey responses for 18 (69%). Their median age was 57 years, 67% were female, 61% had stage IV disease, and 83% had intrahepatic CCA. Prior to pemigatinib, a partial hepatectomy was performed in 44% of the patients, and 66% of the patients received 2–4 prior lines of systemic therapy. All patients were treated with platinum-based regimens as the first-line treatment for unresectable/metastatic disease. The median follow-up time on pemigatinib was 12.6 (range: 2.3–28.4) months, and their median real-world progression-free survival (rwPFS) was 12.1 months (95% CI 7.2-NR). The physician-assessed objective response and disease control rates were 56% and 89%, respectively. For the nine patients who discontinued pemigatinib, the median treatment duration was 10.6 months (range: 0.8–21.7). Disease progression was the most common reason for discontinuation (89%). None discontinued due to adverse events. Conclusions: Objective response rates, disease control rates, and a PFS comparable to that in the phase 2 FIGHT-202 trial was reported with pemigatinib use in this Canadian PSP cohort.

## Simple Summary

Cholangiocarcinoma is a rare and aggressive cancer of the bile ducts. For some patients, this cancer is linked to a genetic change in the FGFR2 protein. In 2021, Health Canada approved pemigatinib as a targeted therapy for patients with previously treated, unresectable, locally advanced or metastatic cholangiocarcinoma with an *FGFR2* fusion or rearrangement. However, there is little real-world data on the use of pemigatinib in these patients in the Canadian setting. This study included 18 patients across six provinces who received pemigatinib through a patient support program. Most had advanced disease, and many had already received several lines of chemotherapy. After starting pemigatinib, over half showed a measurable response in their cancer, and nearly 90% had some level of disease control. On average, pemigatinib delayed disease progression for approximately one year. Importantly, none of the patients stopped treatment because of side effects. These results are comparable to those from earlier clinical trials, suggesting pemigatinib is effective and well tolerated in real-world settings. These findings reinforce the clinical value of pemigatinib for Canadian patients with cholangiocarcinoma and underscore the need for timely access to both targeted therapies and comprehensive genetic testing to ensure patients receive the most effective, personalized care.

## 1. Introduction

Cholangiocarcinoma (CCA) is an aggressive cancer of the bile duct epithelium classified as intrahepatic or extrahepatic based on the tumour location [1,2]. This is a rare disease comprising only 3% of gastrointestinal cancers and diagnosed in ~10,000 people per year in the United States [1,3]. However, the prognosis is poor, with only 20–30% of CCA diagnosed at an early stage and a 5-year relative survival rate of ~10% [2,4,5,6]. For resectable intrahepatic or extrahepatic CCA, the primary treatment is surgical resection with regional lymphadenectomy [7,8]. Depending on the extent of residual disease, patients then proceed to surveillance with or without adjuvant chemotherapy or chemoradiation. Patients with unresectable or metastatic CCA are typically sent for biopsy and molecular profiling and then offered the appropriate systemic therapy, chemoradiation, or locoregional therapy, in addition to the best supportive care. In those with an adequate response to initial treatment, resection may be reconsidered.

The TOPAZ-1 and KEYNOTE-966 trials demonstrated the benefit of adding PD-(L)1-directed immunotherapy to gemcitabine–cisplatin in patients with advanced unresectable or metastatic CCA [9,10]. Currently, gemcitabine–cisplatin ± durvalumab is recommended by both the National Comprehensive Cancer Network (NCCN) and the European Society For Medical Oncology (ESMO) as the first-line treatment for unresectable/metastatic CCA; additionally, the NCCN endorses a combination of gemcitabine–cisplatin with pembrolizumab as an alternative first-line option [7,8]. As the second line, the ABC-06 trial investigated FOLFOX + active symptom control (ASC) vs. ASC in patients progressing on gemcitabine + cisplatin [11]. Said study showed that FOLFOX improved the overall survival (OS) compared with that under active symptom control alone (median OS: 6.2 versus 5.3 months, respectively). In the real-world setting, only 15–25% of second-line patients are fit enough for this option [12]. Comprehensive genomic profiling, including next-generation sequencing of multiple classes of mutations, has helped identify actionable alterations, including in genes encoding fibroblast growth factor receptors (FGFRs), for targeted therapeutic opportunities [2]. *FGFR2* gene alterations are involved in the pathogenesis of CCA, and approximately 10 to 15% of patients with intrahepatic CCA harbor *FGFR2* alterations [1].

Pemigatinib is a selective oral inhibitor of FGFR1-3. Its efficacy in previously treated advanced CCA was evaluated in the open-label single-arm FIGHT-202 trial [13]. This study included three patient cohorts: patients with *FGFR2* fusions or rearrangements (*n* = 107), patients with other *FGF/FGFR* alterations (*n* = 20), and patients without *FGF/FGFR* alterations (*n* = 18). Patients received an initial dose of 13.5 mg orally once daily for 14 days, followed by 7 days off. The primary end point was the objective response rate. With a median follow-up of 45.4 (range: 19.9–53.7) months, 37% of the patients with an *FGFR2* fusion or rearrangement had an objective response, including three complete responses. The median response duration was 9.1 months. Disease control (an objective response or stable disease) was achieved in 82%. The median progression-free survival (PFS) and OS were 7.0 (95% CI: 6.1–10.5) months and 17.5 (95% CI: 14.4–22.9) months, respectively. No patients with other *FGF*/*FGFR* alterations or who lacked *FGF*/*FGFR* alterations achieved a response.

The FIGHT-202 study found that the treatment was generally well tolerated across the cohort. The most common adverse event observed was hyperphosphatemia, which typically occurred early on in treatment (median time to onset: 15 days [95% CI: 8–47]). Management strategies included dietary phosphate restriction, concomitant phosphate binders, diuretics, dose reductions, and/or dose interruptions [13]. Other grade 3 or higher adverse events, occurring in fewer than 7% of the patients, included arthralgias, stomatitis, hyponatremia, hypophosphatemia, abdominal pain, and fatigue. Additionally, 4% of the patients experienced serous retinal detachment due to subretinal fluid accumulation, with most cases being grade 1 or 2, except for one reported grade 3 event.

Largely based on the findings from FIGHT-202, the NCCN updated its Clinical Practice Guidelines for Hepatobiliary Cancers in August 2020 to recommend pemigatinib as a treatment option for patients with unresectable locally advanced or metastatic CCA with an *FGFR2* fusion or rearrangement [7]. In September 2021, pemigatinib received a notice of compliance with conditions from Health Canada for the treatment of adult patients with previously treated, unresectable, locally advanced or metastatic CCA with an *FGFR2* fusion or other rearrangement, as detected using an approved test [14].

As there have historically been sizeable delays in the reimbursement of medications in Canada following Health Canada approval, Incyte Biosciences Canada initiated a bridging access program in December 2021, allowing physicians to prescribe pemigatinib to treat CCA in patients with the approved indications. There is limited literature and real-world information regarding the clinical course of CCA in Canada. Therefore, we aimed to describe the real-world management and outcomes of patients enrolled in this Canadian patient support program (PSP) to receive pemigatinib for unresectable locally advanced or metastatic CCA.

## 2. Materials and Methods

### 2.1. The Study Design and Population

This was a retrospective, multi-site case series of Canadian patients who were prescribed pemigatinib for the treatment of CCA as part of the PSP. Patients enrolled into the PSP were those with previously treated, unresectable, locally advanced or metastatic CCA with *FGFR2* fusions or rearrangements. Included patients were those aged 18 years or older; prescribed 13.5 mg of orally administered pemigatinib once daily (21-day cycle; 2 weeks on, 1 week off); and enrolled in the PSP between January 2021 and October 2022, inclusive. Patients were excluded if they received pemigatinib as part of an interventional clinical trial, received systemic therapy for another primary malignancy after or within 12 months prior to the initiation of pemigatinib, or were never administered pemigatinib. Approval for this study was obtained from the Health Research Ethics Board of Alberta Cancer Committee.

### 2.2. Study Data

Data was collected through an online survey of physicians who prescribed pemigatinib as facilitated by this PSP. With their consent to participate, prescribing physicians were provided a secure survey link via the Qualtrics platform [15]. The prescribing physicians then confirmed the above eligibility criteria of their respective patients and provided patient-level demographics, clinical characteristics, and treatment-related information collected as part of routine care. No information personally identifying the patients was captured during this study, including but not exclusive to their names, dates of birth, social insurance numbers, personal health numbers, and medical record numbers. The data collection proceeded from June 2023 to August 2023.

### 2.3. The Statistical Analysis

The primary outcomes of this study were the physician-assessed, real-world objective response rate (rwORR), disease control rate (rwDCR), and progression-free survival (rwPFS). The rwDCR was defined as the proportion of patients with radiographic evidence of a complete response, a partial response, or stable disease. Descriptive statistics were used to summarize the patients’ demographic and clinical characteristics, treatment patterns, pemigatinib utilization metrics, and clinical outcomes (treatment duration, best radiographic response). Continuous variables were presented as the median with the range or the mean with standard deviation, while categorical variables were presented as the frequency with a percentage. The Kaplan–Meier method was used to assess the rwPFS and the rwOS from the date of treatment initiation while accounting for right-censoring. All analyses were performed using R Studio (Version 2023.03.0+386) [16].

## 3. Results

Survey responses were received for 18 of the 26 patients (69%) who in whom pemigatinib therapy was initiated in the PSP. Their median age was 57 years, 12 (67%) were female, 11 (61%) had stage IV disease at diagnosis, and 15 (83%) had confirmed intrahepatic CCA (Table 1). Prior to pemigatinib initiation, a partial hepatectomy was performed in eight patients (44%). Of the patients receiving a partial hepatectomy, seven (88%) received adjuvant chemotherapy. Seven patients (39%) received adjuvant systemic therapy prior to pemigatinib, and seven patients (39%) received two or more prior lines of systemic therapy in the unresectable/metastatic setting. Capecitabine was the most common adjuvant therapy (57%), while gemcitabine + platinum was the most common first-line unresectable/metastatic therapy (78%). The median length of the prior systemic treatments was 6 cycles (range: 1–8) in the adjuvant setting and 9 cycles (range: 1–35) in the unresectable/metastatic setting.

**Figure 1:**
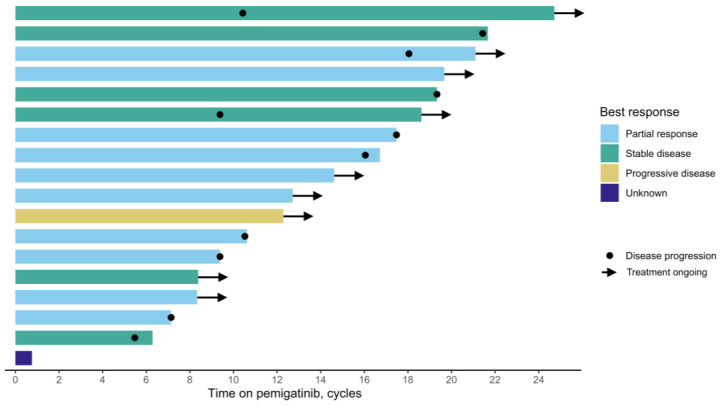
Swimmer plot of pemigatinib treatment duration and best radiographic response.

The median time from *FGFR2* testing to pemigatinib initiation was 5 months (Figure 1, Table 2). The rwORR was 56%, and the rwDCR was 89%. Nine patients (50%) were still receiving pemigatinib at the last follow-up. Among those who discontinued treatment, the median treatment duration was 11 cycles (range: 1–22), with the most common reason for discontinuation being disease progression (89%). One patient stopped treatment due to hospital admission, while toxicity was not reported as a reason for discontinuation.

**Figure 2:**
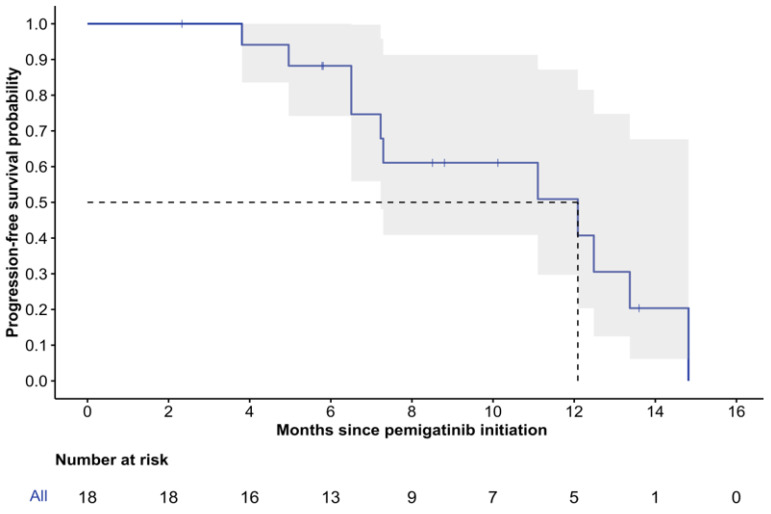
Kaplan–Meier curve of real-world progression-free survival. 95% confidence intervals are depicted in gray; dashed lines illustrate median PFS.

**Figure 3:**
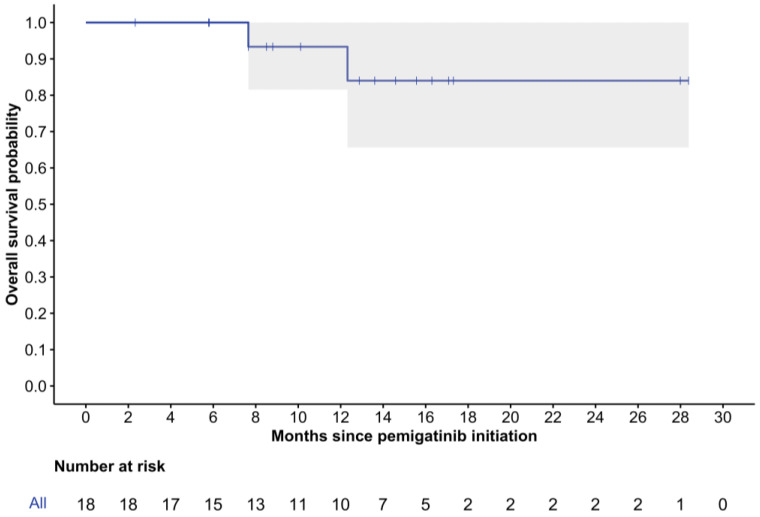
Kaplan–Meier curve of real-world overall survival. 95% confidence intervals are depicted in gray.

The median follow-up time from pemigatinib initiation was 12.6 months (range: 2.3–28.4), with two patients (11%) deceased at the last follow-up. The median rwPFS was 12.1 months (95% CI: 7.2-NR), and the 6-month rwPFS was 88% (95% CI: 74–100%) (Figure 2). While the median rwOS was not reached, the 12-month rwOS was 93% (95% CI: 82–100%), and the 24-month rwOS was 84% (95% CI: 66–100%) (Figure 3).

## 4. Discussion

Through this multi-site case series, we evaluated the real-world use of pemigatinib in Canadian patients diagnosed with locally advanced or metastatic CCA via the PSP. We collected demographic and clinical data for 18 out of 26 patients who initiated pemigatinib in the PSP at the time of the physician survey. This patient cohort was distributed across six Canadian provinces. At a median follow-up of 12.6 months, the rwORR on pemigatinib was 56%, and the rwDCR was 89%. Together, these measures support both the tumouricidal and tumouristatic effects of pemigatinib that were originally demonstrated in the FIGHT-202 trial, with objective response and disease control rates of 37% and 82%, respectively [13]. The clinical benefit of pemigatinib is further reinforced by the median rwPFS of 12.1 months (95% CI: 7.2–NR), compared to 7.0 months (95% CI: 6.1–10.5) in FIGHT-202. Recent observational studies based in the United States and Europe (France and Italy) have also shown similar findings, demonstrating the real-world benefit of pemigatinib in patients with CCA [17,18].

To our knowledge, this was the first study describing the use of pemigatinib for CCA in a Canadian setting. Despite receiving notice of compliance with conditions from Health Canada in 2021 and a positive recommendation by the health technology assessment body in Quebec, INESSS, pemigatinib is currently only available in Alberta and Quebec through case-by-case review [19]. At the time of publication, 17 countries are funding pemigatinib based on the results of FIGHT 202, and numerous biliary tract guidelines recommend pemigatinib as the preferred treatment for previously treated advanced/metastatic CCA with an *FGFR2* fusion or another rearrangement [7,8,20]. *FGFR2* testing for patients with CCA is publicly funded in Ontario, Alberta, and Quebec and is otherwise available via various research programs, highlighting a disconnect between the funding approaches to biomarker testing and their associated therapies. In April 2022, Canada’s Drug Agency, CDA-AMC, formally CADTH, provided a final recommendation of “do not reimburse” for pemigatinib. The main concern from CDA-AMC was the uncertainty of its clinical benefit due to the lack of a comparator arm in FIGHT 202. Pemigatinib was resubmitted to CDA-AMC in October 2024, and in May 2025, pemigatinib received a final recommendation of “Reimburse with conditions” for the treatment of adults with previously treated, unresectable locally advanced or metastatic cholangiocarcinoma with an FGFR2 fusion or another rearrangement. Four additional studies to address the gaps in the evidence were part of the resubmission, of which three studies described the real-world experience with pemigatinib [21]. The results of the study described above were reviewed as part of the resubmission and highlight the importance of the generation of real-world evidence in addressing evidence gaps, in particular for rare cancers where phase III randomized trials may not be feasible.

These findings should be interpreted in the context of several limitations. As an observational study, the exclusion of certain patient characteristics (e.g., biliary drainage or stent placement) during the data collection allowed for potential unmeasured confounding that could not be accounted for in the analysis. Due to the low incidence of CCA and the duration of the PSP, the potential cohort size was capped at 26 patients, and the final cohort size was highly dependent on the physician survey response rate. Our survey methodology facilitated efficient data collection across multiple healthcare jurisdictions, but given a response rate of 69%, there remains the possibility of selection bias, and this nonprobability sampling may limit the generalizability of our analysis to all patients with locally advanced/metastatic CCA in Canada. Information on biliary drainage or stent placement prior to pemigatinib initiation was not collected, and thus we were unable to assess its potential impact on the outcomes.

## Figures and Tables

**Table 1:** Baseline characteristics and treatments received prior to pemigatinib initiation.

Characteristic	N	All Patients, N = 18
Age at initial diagnosis, years	18	
Mean ± SD		53 ± 8
Median (range)		55 (34, 64)
Age at pemigatinib initiation, years	18	
Mean ± SD		55 ± 8
Median (Range)		57 (36, 66)
Sex	18	
Female		12 (66.7%)
Male		6 (33.3%)
Province	18	
British Columbia		5 (27.8%)
Ontario		5 (27.8%)
Alberta		3 (16.7%)
Quebec		3 (16.7%)
Atlantic provinces ^1^		2 (11.1%)
CCA stage at initial diagnosis	18	
I		1 (5.6%)
II		4 (22.2%)
III		2 (11.1%)
IV		11 (61.1%)
CCA subtype	18	
Intrahepatic		15 (83.3%)
Unknown		3 (16.7%)
ECOG PS at pemigatinib initiation	18	
0		3 (16.7%)
1		15 (83.3%)
Surgical procedure received	8	
Partial hepatectomy		8 (100.0%)
Received adjuvant systemic therapy	18	
Yes		7 (38.9%)
Lines of systemic therapy in unresectable/advanced setting	18	
1		11 (61.1%)
2		6 (33.3%)
3+		1 (5.6%)
Systemic therapy regimens		
Gemcitabine + platinum	18	16 (88.9%)
Capecitabine	18	5 (27.8%)
FOLFOX	18	3 (16.7%)
FOLFIRI	18	2 (11.1%)
Other	18	7 (38.9%)
Systemic therapy regimen length, cycles (denominator = 34)		
Mean ± SD		10 ± 8
Median (Range)		8 (1, 35)

Abbreviations: SD, standard deviation; CCA, cholangiocarcinoma; ECOG PS, Eastern Cooperative Oncology Group performance status; FOLFOX, folinic acid/fluorouracil/oxaliplatin; FOLFIRI, folinic acid/fluorouracil/irinotecan ^1^. Atlantic provinces include Nova Scotia and Newfoundland and Labrador.

**Table 2:** Pemigatinib treatment characteristics.

Characteristic	N	All Patients, N = 18
Time from *FGFR2* testing to pemigatinib initiation, months	17	
Mean ± SD		7 ± 7
Median (range)		5 (1, 23)
Best radiographic response	18	
Complete response		0 (0%)
Partial response		10 (55.6%)
Stable disease		6 (33.3%)
Progressive disease		1 (5.6%)
Unknown		1 (5.6%)
Treatment duration, cycles	9	
Mean ± SD		12.2 ± 7.0
Median (range)		10.6 (0.8, 21.7)
Reason for treatment discontinuation	9	
Disease progression		8 (88.9%)
Other		1 (11.1%)
Baseline serum CA19-9, U/mL	14	
Mean ± SD		681 ± 1519
Median (range)		116 (1, 5646)
Follow-up serum CA19-9, U/mL	13	
Mean ± SD		2186 ± 7662
Median (range)		35 (1, 27,685)
Change in serum CA19-9, U/mL	12	
Mean ± SD		1599 ± 6455
Median (range)		−34 (−1509, 22,039)

Abbreviations: SD, standard deviation.

## Data Availability

The data presented in this study are available on request from the corresponding author.

## Abbreviations

The following abbreviations are used in this manuscript:

CCA	Cholangiocarcinoma
PSP	Patient support program
rwPFS	Real-world progression-free survival
CI	Confidence interval
NR	Not reached
NCCN	National Comprehensive Cancer Network
ESMO	European Society for Medical Oncology
FOLFOX	Folinic acid/fluorouracil/oxaliplatin
OS	Overall survival
ASC	Active symptom control
FGFR	Fibroblast growth factor receptor
rwORR	Real-world objective response rate
rwDCR	Real-world disease control rate
SD	Standard deviation
ECOG PS	Eastern Cooperative Oncology Group performance status
FOLFIRI	Folinic acid/fluorouracil/irinotecan
